# Third Sector Organisations’ Contributions to the Health and Care Ecosystem

**DOI:** 10.5334/ijic.9813

**Published:** 2025-08-06

**Authors:** Sanna Tuurnas, Henna Paananen, Anna-Aurora Kork

**Affiliations:** 1 Faculty of Management and Business, Tampere University, Finland; 2 School of Management, University of Vaasa, Finland

**Keywords:** health and care ecosystems, value contribution, co-production, third sector organisations, integrated care

## Abstract

**Introduction:**

In integrated care models, third sector organisations (TSOs) are essential but often undervalued parts of the health and care ecosystem to address care fragmentation and inequity of access. In this study, we illustrate the value contributed by TSOs in a co-production setting.

**Methods:**

In this qualitative study, frame analysis was used to explore the varied interpretations of TSOs’ value contributions at different levels of the ecosystem. The qualitative interview data were collected from 16 informants in the context of a Nordic welfare state, Finland. The interviewees represented a variety of health and care TSOs.

**Results:**

The resulting frames were seen as organising principles that are socially shared. The analysis revealed a threefold interpretative schema of TSOs’ value contributions: strengthening the ideals of civil society, advancing the public service system and connecting people to communities.

**Discussion:**

The findings demonstrate that TSOs contribute values in health and care ecosystems at all levels—micro, meso and macro—by integrating short- and long-term policy objectives, ensuring that their actions result in meaningful and sustainable benefits across all levels of the public service ecosystem.

**Conclusions:**

This study underlines the need to integrate civil society into the health and care ecosystem and, specifically, to acknowledge the holistic contribution of TSOs when developing integrated care models. The key contribution of this study is demonstrating the diverse ways in which TSOs can contribute value for integrated care.

## Introduction

Third sector organisations (TSOs) play are crucial actors as intermediaries between citizens and public service organisations within complex health and care systems. They generate collective benefits, such as social support, community cohesion and enhanced volunteer employability [1,2,3,4]. In integrated care models, TSOs have a crucial but often underappreciated contribution for addressing care fragmentation and inequity of access [4].

Overall, TSOs can be seen as agents of the non-profit sector, the non-governmental sector or the social economy. While these definitions overlap, this article uses the term ‘third sector’ to highlight the unique position of TSOs in addressing citizens’ needs alongside the market and public sector, while specifically advocating for their reference groups. They often take responsibility for the most vulnerable citizens, acting as their advocates [5].

Following Wankah et al.’s [6] conceptualisation, integrated care aims to foster collaboration across organisational and professional boundaries to deliver cohesive care to patients, families and caregivers within their communities. This study approaches integrated care from the perspective of TSOs in delivering health and care. Despite the growing interest in collaborative, intersectoral models in the health and care sectors, the contribution and position of TSOs in these models remain underexplored. Public service organisations do not consistently acknowledge the value of TSOs in the health and care ecosystem [6]. The concept of ecosystems offers a robust framework for analysing the contributions of various actors—including TSOs, health and care professionals, citizens and policymakers—to societal value through their interactions in service delivery. At the same time, policy rhetoric recognises the need to integrate civil society actors—both as individual co-producers and volunteers, and as their organised forms, such as TSOs—in multisectoral collaboration for better outcomes and societal value [see e.g., 7,8]. However, the performance of collaborative efforts may be more difficult to acknowledge [9,10]. Assessing the value of multistakeholder initiatives requires multidimensional and holistic approaches to demonstrate the broader societal impact of TSOs [1]. In light of this, the central question of this article emerges: *How do TSOs contribute value to the health and care ecosystem?*

This study examines TSOs’ value contribution within public service systems, specifically in health and care ecosystems. Our purpose is to understand how TSOs contribute to the co-production of public service delivery, enhancing citizen welfare and utilising experiential knowledge to improve the value of the public service ecosystem [12]. The key contribution of this study lies in demonstrating the diverse ways in which TSOs can support the values of integrated care.

### Conceptual background

A service ecosystem is defined as a relatively self-contained, adaptive network of resource-integrating actors connected by shared institutional logics and engaging in mutual value creation through service exchange [13]. Through collaboration within this interconnected system, value accumulates, fostering innovations and outcomes that benefit all participants and contribute to the ecosystem’s overall resilience and growth [12].

Health and care ecosystems form a special type of ecosystem where different logics—from business logic to patient-care logic—coexist [14]. The community logic of civil society organisations adds another type of institutional logic to the collage of diverse and competing logics within the ecosystem. A care ecosystem uses a similar approach but is focused more on composition. It refers to the constellation of diverse actors involved in new forms of care delivery across organisational boundaries [15]. However, for the sake of clarity, we use the concept of health and care ecosystems in this study. The main concept of ecosystem thinking involves the integration of resources across various levels and actors within the ecosystem—specifically, at the micro, meso and macro levels [12]. Adopting an ecosystem approach facilitates understanding of the value of TSOs in fostering collaborative and participatory processes within this interconnected structure and, through that, contributing value to the wider ecosystem. Recognising the interconnectedness of these levels and actors is essential for understanding TSOs’ value contributions within public service systems [16]. In such ecosystems, value cannot be measured simply by summing individual actors’ contributions. Instead, a broader view is needed that considers the collective value derived from collaboration, social impact and the dynamic interactions among actors [16,17].

For this purpose, we use the concept of co-production as a core instrument for collaborative models [18]. Here, we refer to co-production especially as comprising activities between TSOs and public service organisations. TSOs exercise strong agency as essential nodes connecting citizens and public service organisations, generating collective benefits such as social support, community cohesion and volunteer employability [4,19]. Co-production can be used as a means of facilitating the achievement of the underlying values of integrated care as a collaborative or shared responsibility within the service ecosystem [20]. Lindsay et al. [4, p. 152] recognised TSOs’ essential contribution for co-production as due to their rootedness and responsiveness to user groups and communities, which ‘has led to a growing consensus that the third sector can potentially play a key role in fostering co-production’. However, the contribution of TSOs to co-production is not clear; they have previously been seen mainly as organisational partners in public service production [21,22], but we argue that they may also have another kind of value—as mediators in co-production between public bodies and individual citizens due to their ability to give voice to citizens and to empower them [4].

Ewers and Evett [23, p. 78] highlighted the importance of civil society actors in fostering meaningful co-production, noting their ability to offer co-producers ‘sufficient entrepreneurial space’ and to draw on their community-driven knowledge. Martin [21] emphasised that TSOs’ facilitation of meaningful co-production gives them the potential to both strengthen their connections to their reference groups and reinvigorate their advocacy function in general. This potential prompts an extended examination of TSO activity in co-production beyond the direct citizen–TSO interface towards a complete public service ecosystem approach. Many countries have drafted institutional guidelines for promoting such an approach. For example, in the UK, integrated care systems have a statutory duty to align and work with TSOs [24]. Moreover, the Italian Third Sector Reform 2017 promotes co-production by recognising the value of TSOs in delivering public services and encouraging their collaboration with public authorities [25], to name a few examples.

Overall, in this study, we aimed to broaden the understanding of TSOs’ contribution to the public service ecosystem by holistically framing their value beyond merely economic value [9,10]. This framing demonstrates their accomplishment of their social mission and the benefits it brings to individuals, communities and the environment within collaborative settings [26].

## Methods

### Setting

Our qualitative research followed an interpretive approach, guided by a research team skilled in co-production, frame analysis methodology, and health and social management. Researcher triangulation and collaborative discussions ensured reflexivity throughout the analysis and writing process. The empirical data were collected in Finland, a Nordic welfare state where TSOs have made essential contributions for the national health and care systems especially due to their expertise in providing services to special service user groups, including marginalised groups [27]. In Finland, the integration of health and care services has been one of the key targets in reforming the system to enhance equity. However, there are challenges in the coordination of these services, both across different user groups and between regions [28]. Overall, the Finnish health and care systems have been described as fragmented, leading to inequalities, particularly in access to care. According to Tynkkynen et al. [29], a higher proportion of individuals in Finland reported unmet care needs compared to those in Sweden. Additionally, the Finnish welfare system continues to depend heavily on trained public servants despite the critical shortage of professionals in social and health care worldwide, including in Finland [30]. These challenges underline the need for integrated care models in the country [31].

While TSOs have traditionally acted as key service producers and innovators in the Finnish health and care ecosystem, their value as part of integrated care models may not necessarily be institutionalised. This is despite the fact that they also act as nodes in the complex service processes by addressing the gaps in the service system and supporting services [32,33]. Moreover, as in many other countries, the value contribution of TSOs in service ecosystems has changed over the years, reflecting the international New Public Management trends that emerged in the 1990s [34]. These trends promote a ‘doing-more-with-less’ ideology, emphasising resource efficiency while promoting greater reliance on citizen and civil society contributions through encouragement policies [35]. Through these policies, TSOs are recognised as partners in governance, fostering active citizenship and volunteerism.

### Participant recruitment

The data were derived from interviews of 16 key representatives of TSOs operating in the field of health and social services in the Pirkanmaa region of Finland. The informants were recruited based on their involvement in stakeholder groups preparing for the national health and social services reform. They were assumed to possess natural, in-depth knowledge of the value contributions of TSOs in society [36] and were actively engaged in collaboration with public organisations.

Additionally, the selected TSOs reflect the wide range of health and care providers in Finland, which are characterised by different institutional and financial positions, scopes of operation and operating models [see Table 1]. In Finland, many TSOs deliver health and social services while undertaking advocacy work at different governance levels—ranging from individual service organisations to local and regional policy-making organisations, and to organisations participating in national legislation processes. Some TSOs operate locally on a voluntary basis, focusing on community-based issues in neighbourhoods, whereas others operate at a regional or national level with better resources that allow them to hire experts to support a large pool of volunteers.

**Table 1:** Characteristics of third sector organisations involved in this study.


INTERVIEWEE	RANGE OF TSO OPERATIONS	OPERATIVE SCOPE OF THE TSO	SERVICE PROVISION

Manager	National	General citizen participation and empowerment, community approach	yes

Planning officer	National	Patient organisation, health promotion	yes

Manager	National	Mental health promotion and advocacy	yes

Manager	National	Multifaceted volunteering functions for social welfare and health promotion	yes

Specialist	National	Support and advocacy for disabled persons	yes

Specialist	National	Patient organisation, health promotion	no

Manager	National	Support and advocacy for families with special needs	no

Manager	National	Support and advocacy for families with special needs	no

Planning officer	National	Health and social welfare promotion and advocacy for young people	no

Specialist	National	Support and advocacy for disabled persons	no

Manager	Regional	Patient organisation, support for families, support for research	yes

Group interview: Manager, submanager, counsellor	Local	Inequity prevention, community approach	no


This study was conducted according to the ethical guidelines of the Finnish National Board on Research Integrity for studies involving human participants. The participants were informed about the study and their rights, and their informed consent was obtained in written and orally before the start of the interviews. The interviews were semi-structured thematic interviews. This method offers a balance between structure and flexibility, allowing an in depth exploration of topics while still following a general guide, thus helping to uncovering rich, detailed information that might not emerge in a fully structured interview [37]. The interview topics consisted of the mission of TSOs, the role of TSOs in reforming services, the position of the TSO in the health and care ecosystem and collaboration, and the TSO’s visions and expectations for future health and care systems concerning their own evolving role within it. The interviews were conducted in person and lasted approximately 60 min each. We audio-recorded, transcribed verbatim and anonymised the interview data. The interview text excerpts in this article were translated into English from Finnish by the authors and were assigned identification numbers (IDs) to de-identify them.

### Identification of frames of TSO’ value in co-production

Before we analysed the interview data, we specified how the frames—text or speech with multiple elements that formed a pattern—would be identified. The frames were identified by analysing the speech patterns and clusters [38] that described TSOs’ actions and contributions in the public service ecosystem. The resulting frames are seen as TSO organising principles that are socially shared, have endured over time and symbolically shape the social world [39].

The analytical concept of a frame was drawn from the work of Erving Goffman [40] to analyse micro-level social interactions and human experiences [41]. As interest in it grew, it was also used in policy analysis [e.g. 42,43,44], as well as in management and organisational literature [45]. Frame analysis has since undergone widespread adaptation and multiple applications. However, its central tenet remains that conceptions are shaped not primarily by events and actions but by how these are interpreted [43] and by the tacit understandings constructed through social interaction [41]. For example, actors’—such as TSO employees’—responses to the question *What is going on here?* in co-production affect the conception and interpretation of events alongside the solid facts [41,43]. The frame adapted by an actor reveals what they see as relevant to the activity in a certain context. Frame analysis usually has two analytical tasks: help uncover the construction of meaning in certain contexts and make it possible to identify the frames’ effects [42].

### Data analysis process

**Figure 1:**
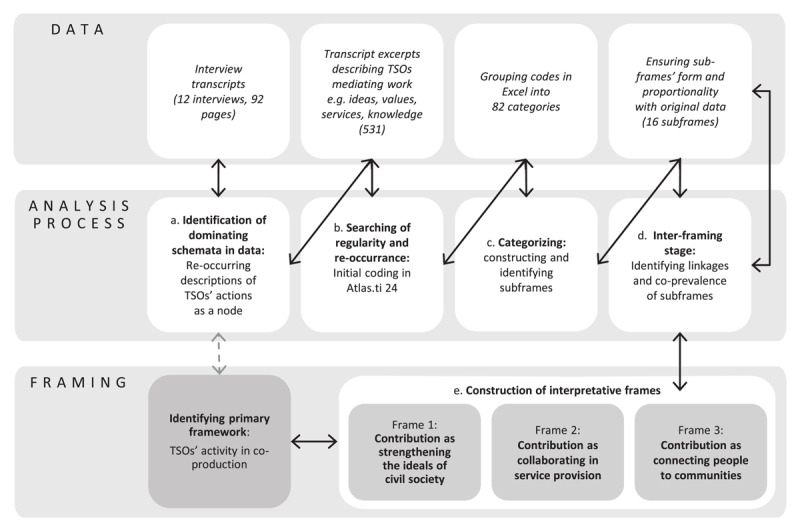
Description of this study’s analysis process.

Our frame analysis was aimed at identifying frames that would clarify the meaning of co-production for TSOs and their value contribution to such co-production. The analysis consisted of the following five stages with coding and categorising. Coding phase was conducted with Atlas.ti 24 software and further categorizing in Windows Excel. (Figure 1).

a) The analysis began with the construction of a primary framework for this article to identify TSOs’ contribution into co-production The data were first read carefully, and then, the dominating schemata were identified. The data featured recurring descriptions of TSOs’ positions and actions as mediators of various elements, including ideas, values, services and knowledge, between public health and care organisations and the ‘outer world’. A total of 531 transcript excerpts met the selection criteria

Sample text excerpt: *I see our greatest task as [to] reveal that people, as citizens, can do well. Maybe that’s the thing. Maybe it basically comes from human dignity and fundamental rights. [It’s about] the UN Convention of the Rights of People with Disabilities, which Finland has ratified—how rights of disabled people are described in that agreement and what we have committed to*.

b) The selected transcripts, which described TSOs’ contributions to health and care ecosystems, were further examined to find descriptions of their aims—whether to promote something, represent someone or stand for a cause in the health and care ecosystem. Aspects related solely to health and care system reform were excluded. Then, the included descriptions were assessed for regularity and recurrence before being coded. Initial codes were developed to indicate the excerpts’ subjects. Then, these excerpts were grouped into 82 initial categories, guided by these analytical questions: *What is being promoted? What does it stand for? For whom is it?*

Sample code: *Work aimed at upholding the fundamental rights of disabled people*

Sample initial category: *For whom and what TSOs stand for – Fundamental human rights*

c) The categorisation of the excerpts continued with the construction of 16 subframes. During this stage, the research team members repeatedly reflected on the identified subframes, comparing them with the original data to ensure their accuracy and proportionality.

Sample subframe: *Promoting a holistic perception of citizenship*

d-f) At this interframing stage, the linkages and co-prevalence of the subframes were analysed to construct three frames representing TSOs’ value contribution. To ensure the trustworthiness of the data analysis, these interpretative frames were compared, discussed among the authors and aligned with the primary framework established in the first stage.

Sample frame: (Frame 3) *TSOs’ value in strengthening the ideals of civil society*

## Results: Frames of TSOs’ value contributions to co-production

Our data analysis identified three value contributions of TSOs to co-production: strengthening civil society ideals, advancing the public service system and fostering connections between people and communities. Each of these contributions is defined and contextualised by subframes (Figure 1).

### TSOs’ value in strengthening civil society ideals

Value in co-production is broadly construed at the societal level as benefiting society as a whole. In this context, TSOs contribute value through their ability to mobilise individuals and communities. They function both as channels and catalysts of civic activity, serving as platforms for different forms of citizen participation.

This framing underlines TSOs’ societal value as active participants in shaping and stimulating societal discussions on their causes and their members’ positions. TSOs conduct their advocacy both locally and nationally, and some of them alternate their scopes depending on the circumstances. As only a few TSOs have a large organisational structure and multiple hired professionals, the strength of their advocacy primarily stems from their members and their interorganisational efforts.

*We do a lot of influence work at the county level, which we will continue through collaboration, emphasising [the need for peer support and special target group knowledge] for a stronger front.’ (ID 10)*

The value of the entire third sector is framed by this ability to activate civil society and to garner support for individuals from different communities. TSOs’ representative contribution is framed around the interests and needs of their members and target groups. Their widely shared mission is to ensure that these voices are heard in discussions of general societal issues and more specific issues. The TSOs described promoting fundamental human rights that might be contested—for example, the right of disabled individuals to participate in democratic decision-making or to conveniently access public spaces. TSOs represent a holistic approach to humanity and are eager to steer public discourse in that direction.

Few other actors extend their services to people in need at the grassroots level. This operating logic sets them apart from other service and support systems based on citizen activity.

*Our mission is to support citizenship and encourage people by asking, ‘How can you participate in this society?’ Participation is an important issue here. Our members need support, for instance, in understanding how to participate in decision-making in society, how to vote, how to formulate one’s own opinion and how to find information presented in plain language so they can understand an issue. For example, we have always organised ‘shadow elections’ before the real elections, be it presidential or local government elections…. (ID 9)*

Additionally, this value is linked to optimising scarce societal resources. Without this contribution of TSOs, market and efficiency-driven logics could dominate the public sphere.

### TSOs’ value in advancing the health and care system

The second frame of TSOs’ value in co-production relates to their contribution to the health and care system. At first glance, their value within the public service system can seem self-evident. However, according to our analysis, framing this value is more multifaceted than simply describing TSOs’ participation in health and care service production. TSOs work within the system by voicing the needs of their user groups and creating pressure for service development through advocacy. These insights are found by inspecting service chains from the viewpoint of the TSOs’ members and target groups. Service system guidance is seen as a particularly effective activity and involves TSOs helping their members navigate highly fragmented service structures. In addition, TSOs align their own activities to fill gaps in services and even produce services themselves.

*One aspect that has been lacking [in services] is this kind of psychosocial support […]. So far, the main thing has been arranging doctors’ or nurses’ appointments. But in the future, we will focus more on psychosocial support. (ID 2)*

*Yes, our agenda is also to get a place for peer support in the service paths. (ID 6)*

Co-production affects the health and care system by developing partnerships, collaborative practices and the service system itself. Co-producing services can improve the wider system by enhancing understanding of user needs and generating additional support for service delivery from TSO actors. The respondents expressed this specialisation of TSOs and their response to their users’ needs, especially in health services.

Alongside TSOs’ service system development work, in this frame, their value is connected to health promotion work. They address the information needs of the broader public concerning certain diseases or disorders through publicity campaigns. As part of this work, they organise disease prevention activities. These kinds of activities are extremely vital for health and care service systems that struggle with growing financial issues and workforce crises.

*Prevention is something that has been more and more on our agenda. We aim to prevent disease X, as there is much that people can do themselves in order to avoid getting disease X. (ID 4)*

Many TSOs also organise follow-up training for healthcare professionals and produce information for them on highly specific topics, such as the housing needs of disabled persons. Another form of knowledge for supporting professionals is mediated by the experiential knowledge of certified lived-experience workers, drawn from their roles as service users. TSOs facilitate the integration of lived-experience workers within the system. Additionally, TSOs seek to elevate the status and practice of peer support and volunteer work by coordinating the efforts of volunteers and clarifying their value contribution within the service system.

*I think there has been a lot of talk about experts by experience, meaning that those who have been through a certain illness or life situation can give coherent insights about it…. I think it is important to strengthen the role of these actors. (ID 3)*

In this study, the TSOs seemed to be actively developing the service system by introducing new actors alongside established professions. In general, the TSOs framed this particular value contribution as recognising and strengthening citizen’s roles. TSOs educate professionals to serve special groups and promote citizen-centredness in the system. They frame their contribution as having a more legitimate and collective-based impact on the health and care system compared to that of single patients, for instance, who are selected to represent all patients in official patient councils (see [45]).

TSOs’ co-production with public actors’ networks may shift their organisational mission from one-way advocacy work to reciprocal and shared knowledge creation with professionals and citizens, thus highlighting their social importance in co-production.

### TSOs’ value in connecting people to communities

The TSO representatives framed their communally and individually constructed values by connecting their individual experiences and advantages to a wider, community-level perspective. Within this frame of ‘connecting people to communities’, our data revealed that a TSO’s value arises from activities that strengthen its members’ sense of belonging and engagement within their community, and, more broadly, within society. In co-production activities, TSOs can highlight the voices of marginalised civil society members by conveying their messages to public service organisations and policymakers. One way of doing so involves training people to become what are known as *experts by experience*. Examples of these are former substance users who can speak personally about substance use in public service development processes. The TSO representatives in this study noted that such an activity can empower these experts by enabling them to progress, educate others and participate in TSO initiatives. This educational task is framed as verifying the quality of lived-experience work and peer support, as well as acknowledging TSO members’ experiences as valuable knowledge resources and sources of self-esteem.

Overall, TSOs represent themselves as voices of the silent and silenced. This group includes substance users, family members of the mentally ill and other socially excluded groups of citizens. A TSO can offer meaningful activities for these and other marginalised groups, such as organising easy-access hobbies, which enhance their well-being. These activities also build a bridge between people and bond them to their communities. As one community-based TSO representative explained:

*We have quite a large group of those people who have not had any hobbies in their lifetime. Many here say that, for the first time, they have found their own group to which they feel they belong. For the first time, they have found friends…. We have been able to reach this crowd who would not have found their place otherwise. (ID 1)*

TSOs arrange encounters between people in similar life situations and, thus, overcome socially constructed barriers in society. They support individuals by attaching them to a community. Offering them a peer-support group encourages them to share their lived experiences, learn from their peers and gain the support needed to adapt to different social situations or health conditions. The core idea of this approach is to promote individual well-being through a community-driven model that manifests the value and impact of TSOs in co-production.

*We empower people through the community; in [place X], we do activities that strengthen the sense of inclusion and coping skills of disabled people. We do this with a positive approach and encouragement: ‘Hey, you are very good at this, and you can do this.’ (ID 9)*

In this ‘community’ frame, TSOs’ impact is also framed as the support they provide in different life situations. They strengthen citizens’ general life skills during co-production while also bolstering their coping mechanisms for unexpected challenges. Examples of TSOs’ value contribution in this frame are first-hand support for those who have recently discovered an illness and long-term adjustment training to address illnesses and disabilities. TSO actions can aid both the people directly affected and their families, who are often excluded from public service system support mechanisms. TSOs can also enhance their members’ practical skills and physical abilities in rehabilitation.

**Figure 2:**
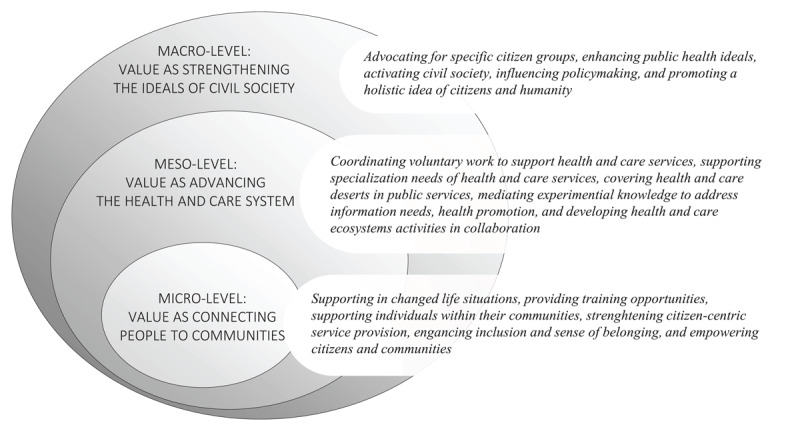
The frames of TSOs’ value contribution to co-production.

To outline our findings, the previously presented frames form a threefold ensemble of TSOs’ value contribution to co-production (Figure 2). Each of these frames offers a lens through which to reflect on TSOs’ contribution in the public service ecosystem at various levels.

## Discussion

In this study, we used co-production as our analytical concept. We distinguished 20 different subframes of TSO work, from which we extracted three frames relating to TSOs’ values in connecting people to communities, advancing the public service system and strengthening civil society ideals. These results indicate that TSOs are essential actors in the health and care ecosystem, as they supplement public services, concurrently specialising in and advancing health and care services.

At the individual level, their value is in creating opportunities for citizens to participate in societal activities—for instance, by coordinating the work of experts by experience. The value of TSOs in co-production is especially visible from the viewpoint of their contribution as acting nodes in the health and care ecosystem, as they complement service provision, are citizen advocates in societal-level debates and assist in policy formulation. Thus, their value contribution seems to accumulate at multiple levels of the ecosystem.

Concerning integrated care, the detected frames are interconnected in many ways. For instance, TSOs’ influence on policymaking could also be seen as a feature of their socio-economic value at the community level. Overall, TSOs create value by integrating short- and long-term objectives, ensuring that their actions result in meaningful and sustainable benefits across all levels of the health and care service ecosystem.

The main contribution of this study is its improvement of the understanding of the multiple ways in which TSOs’ value in co-production is represented. As this study shows, TSOs have great potential to strengthen the integration of care and empower civil society at the societal, organisational and community levels of this ecosystem. This study suggests that TSOs can have a strong agency within health and care ecosystems, but their value is typically strongest in the gaps in the service system and, therefore, may remain hidden. TSOs are community-based organisations that act as essential nodes between citizens and public service organisations, and create value by generating collective benefits, such as increased social support and inclusion, community cohesion, volunteer employability, organisational activities and service provision responsibilities [2,3,4,19].

In collaborative settings, however, performance management is a complex task [10,11]. While this study did not focus on performance management, it calls for a broader understanding of impacts in service ecosystem settings. From the value creation perspective, undertaking outcome-based commissioning, public procurement or establishing partnerships requires a holistic approach [3] that considers the operational logic of different organisations and their capacity to act effectively at the individual, service system and societal levels. Consistent with previous research [3, p. 47], this study shows that value is more than the economic value of short-term outputs.

We suggest that governments should deepen the scope of their collaboration with TSOs and seek softer forms of collaboration than merely outsourcing services to them. Second, regarding implications at the public service organisation level, the full spectrum of TSOs’ value should be considered in co-production activities. If TSOs are seen merely as service providers rather than as co-managers of services [48], governments risk losing a considerable volume of the societal value they provide. Finally, for community-level implications, the ecosystem settings of TSOs can create opportunities to leverage citizen knowledge in developing civil society.

From an international perspective, it should be noted that collaboration with TSOs is perceived differently between countries and welfare state regimes, as well as between diverse policy fields [46]. As the previously mentioned examples from Italy and the UK suggest, there may even be institutional requirements to include TSOs in integrated care models or, at least, to strongly advocate for their involvement.

## Conclusions

In this study, we examined how TSOs contribute value to the health and care ecosystem. The findings emphasise the importance of recognising the holistic approach of TSOs in developing integrated care models. TSOs not only support but also actively foster integration, facilitating a shift in health and care service models towards more cohesive and community-centred approaches [5]. Unlike traditional health and care organisations, which primarily operate within institutional and organisational frameworks, TSOs engage directly at the community level, making integration their core operational logic.

TSOs contribute across multiple dimensions of integration: horizontal, vertical and temporal. This comprehensive integration positions them as unique actors in the ecosystem, distinct from health and care organisations that often remain constrained by siloed practices [31]. TSOs function as vital nodes within the ecosystem, bridging diverse stakeholders and fostering connections that extend beyond conventional institutional boundaries.

This study indicates a need for multi-stakeholder models that embrace ecosystem approaches, incorporating both conventional partners and non-conventional partners, such as TSOs. By integrating communities into health and care ecosystems, TSOs enable citizens to take on active roles as contributors to different types of activities—ranging from their own services to those benefiting others, as well as policy-level advocacy. However, achieving this requires a fundamental shift in mindset and a reconfiguration of professional practices to foster greater openness and collaboration.

From a societal perspective, TSOs are indispensable partners in generating value that transcends traditional service delivery. Their community-oriented, value-driven approaches ensure that services are not confined to a product-oriented scope but are instead aligned with the broader well-being of individuals and communities. This positions TSOs as critical enablers of integrated, inclusive and sustainable health and care ecosystems.

### Implications

This study highlights the value-driven approach to integrated care [19]. Zonneveld and et al. [20] detected over 20 values underlying integrated care. The most commonly identified values were ‘collaborative’, ‘coordinated’, ‘transparent’, ‘empowering’, ‘comprehensive’, ‘co-produced’, and ‘shared responsibility’ and ‘accountability’. Strokosch and Roy [47] proposed a holistic view of the interconnectedness between different domains within the health and social care ecosystem, emphasising the importance of structural, institutional and relational integration. They argued that recognising the unique and shared contexts of various actors and institutions involved in the ecosystem is crucial for the successful integration of health and care models.

Our findings in the present study support this argument by highlighting TSOs as crucial partners in generating societal value, as they extend their services beyond the mere product scope through a value-driven, community-oriented approach. Their focus on addressing societal needs positions them holistically as indispensable actors in fostering well-being and addressing complex social challenges.

For future voluntary- and community-sector studies, the concept of health and care ecosystems offers a robust framework for analysing TSOs’ contributions to societal value. The present study shows that ecosystems provide a valuable theoretical lens through which to explore interdependencies and collaborative efforts that contribute to the creation and enhancement of value beyond traditional organisational and professional boundaries. This perspective encourages an integrated understanding of the contributions offered by diverse stakeholders, including TSOs, in achieving shared goals. However, with power, influence and resources predominantly concentrated within traditional hierarchies, health and care professionals may perceive TSOs as well-meaning but lacking the necessary skills [48]. At the same time, public governments are shaping the role of TSOs as more service producers than citizen advocates. Thus, in TSOs’ collaboration with public organisations to offer services, there is a growing emphasis on professionalisation and market orientation. This shift may sometimes come at the expense of the civic values that underpin TSOs, related to their unique position and community approaches [21,49,50].

The methodological significance of the present study is its introduction of frame analysis for exploring the diverse ways in which TSOs can facilitate the values of integrated care, such as engaging communities, connecting people and helping them navigate service systems. To comprehensively understand value contributions, there is a pressing need for holistic approaches underpinned by qualitative enquiry. Frames, as analytical tools, are particularly effective in recognising, defining and articulating the diverse forms of value generated within ecosystems. They are especially relevant for academics, but also for policymakers. In health and care ecosystems, it is crucial for all actors—including TSOs, health and care professionals, citizens and policymakers—to understand the potential value created by their interactions and contributions.

Furthermore, health and care practitioners and managers can utilise these frames to design and implement strategies that foster collaborative value creation. As part of performance evaluation, frames offer a valuable means of representing collective assessment models. TSOs can leverage these frames to align their objectives with broader ecosystem goals, thereby ensuring that their contributions are recognised and optimised. Demonstrating achievement of organisational goals and wider socio-economic impacts [51] can be useful to funders but can also be a learning opportunity for TSOs regarding their social value in the service ecosystem [40]. Accordingly, the contributions of TSOs can be seen as strategic organisational actions embedded in their social mission of creating value for key stakeholders and society. Thus, the value of these actions includes both the short-term results of the TSOs’ work and its longer-term activities (i.e., its chosen methods and strategies) aimed at influencing service development, policies or society.

Future research should build on these foundational models by incorporating deeper insights from a broader array of ecosystem actors, including citizens, health and care professionals, and policymakers, to enrich the understanding of how value is co-created and sustained within ecosystems. Doing so could enhance the theoretical and practical tools available to TSOs and other stakeholders, ultimately enabling more effective and impactful societal contributions.

Overall, strong integration of various ecosystem actors for better outcomes is echoed previous research related to this topic. As the literature review study of Conquer et al. [52] suggested, effective co-production in integrated care requires a well-defined process that emphasises person-centred design, innovative planning and collaboration. The study highlights that co-production can significantly impact the design and transformation of integrated care services by involving service users, unpaid carers and staff at a higher level of engagement.

There are several inspiring international examples of this approach. A widely referenced example is the Buurzorg Model from the Netherlands—a community-based approach to nursing care that involves nurses working closely with patients and their families to develop care plans tailored to the specific needs of the community [53].

Finally, it is essential to ask how governance can support TSOs within integrated models. To foster their integrative logic and unlock their potential, it is essential to recognise the unique value that they bring to collaborative efforts. However, sustaining their involvement requires that they be provided with financial security and a stable institutional position within the health and care ecosystem. This entails shifting their role from the periphery to the core, ensuring that they are fully integrated into policy- and decision-making processes.

### Strengths and limitations

This study explored TSOs’ contribution to health and care ecosystems using a qualitative approach, which limits the generalizability of the results. However, although the interviews were conducted within a specific Nordic context, the findings offer relevant insights from some of the world’s most advanced welfare states. These implications could be beneficial for countries developing integrated care models, particularly in the delivery of health and social welfare services. The trustworthiness of this study was strengthened through reflexivity and detailed contextual descriptions. Finnish TSOs’ challenges are similar to those in many other countries, such as their non-recognition as key contributors to the co-production of public services, their cost constraints and their experience of reduced outsourcing due to public austerity policies. Despite the difficulties in conceptualising the impact or value of TSOs within service ecosystems, our in-depth frame analysis offers a promising method and perspective for framing TSOs’ diverse contributions at the micro, meso and macro levels.
